# Proposal for a New Histological Scoring System for Cartilage Repair

**DOI:** 10.6061/clinics/2018/e562

**Published:** 2018-11-16

**Authors:** Maria Clara Ponce, Alessandro Rozim Zorzi, João Batista de Miranda, Eliane Maria Ingrid Amstalden

**Affiliations:** I Faculdade de Ciencias Medicas, Universidade Estadual de Campinas (UNICAMP), Campinas, SP, BR; II Departamento de Cirurgia Ortopedica, Faculdade de Ciencias Medicas, Universidade Estadual de Campinas (UNICAMP), Campinas, SP, BR; III Departamento de Ortopedia e Reumatologia, Hospital Israelita Albert Einstein, Sao Paulo, SP, BR; IV Departamento de Patologia, Faculdade de Ciencias Medicas, Universidade Estadual de Campinas (UNICAMP), Campinas, SP, BR

**Keywords:** Cartilage, Histology Scoring System, Regenerative Medicine, Tissue Engineering

## Abstract

**OBJECTIVE:**

This study aimed to develop a new histological scoring system for use in a partial-thickness cartilage repair animal model. Although previous papers have investigated the regeneration of articular cartilage, the good results achieved in small animals have not been replicated in large animal models or humans, possibly because of the frequent use of models with perforation of the subchondral bone plates. Partial-thickness lesions spare the subchondral bone, and this pattern is the most frequent in humans; therefore, new therapies should be tested using this model. However, no specific histological score exists to evaluate partial-thickness model results.

**METHODS:**

Histological sections from 30 ovine knees were reviewed to develop a new scoring system. The sections were subjected to H&E, Safranin O, and Masson's trichrome staining.

**RESULTS:**

This paper describes a new scoring tool that is divided into sections in detail: repair of tissue inside the lesion, cartilage around the lesion and degenerative changes at the base of the lesion. Scores range from 0 to 21; a higher score indicates better cartilage repair.

**DISCUSSION:**

Unlike existing tools, this new scale does not assign points for the positioning of a tidemark; we propose evaluation of the degenerative changes to the subchondral bone and calcified cartilage layer. It is necessary to remove the whole joint to access and study the evolution of the lesion as well as the surrounding tissue.

**CONCLUSION:**

This article emphasizes the importance of a partial-thickness animal model of cartilage repair and presents a new histological scoring system.

## INTRODUCTION

Articular cartilag*e* is tissue that covers the surface of bone joints. It absorbs mechanical impact and facilitates movement by decreasing friction and protecting the bones. It has a firm consistency but is elastic (resilient) and is not vascularized or innervated. It is composed of water, cells (chondrocytes) and extracellular matrix (ECM) and is organized in four layers: superficial, middle, deep and calcified tissue. The calcified layer is firmly attached to and difficult to separate from the subchondral bone. Each layer has various chondrocyte shapes and a different ECM composition (quantity of proteoglycans and disposition of collagen type II). Chondrocytes are responsible for the production, organization and maintenance of the ECM, the quality of which is fundamental for cartilage function 1,2.

If the cartilage is injured, it will not heal properly because it has few stem cells to substitute for lost chondrocytes. The ECM avoids migration, and the lack of vascularization makes the recovery process more difficult. Indeed, a cartilage defect can predispose an individual to osteoarthritis (OA) 3,4. To avoid such a result and to improve cartilage repair, many experimental studies have explored new surgical techniques and even tissue-engineering techniques 2,5,6.

Two experimental surgical models exist. The total-thickness model creates a deep puncture through the cartilage, damaging the subchondral bone and allowing bone marrow cells and blood vessels to migrate to the defect site. In this manner, the intervention is influenced by these cells and can evolve with the formation of intra-lesional osteophytes 7,8. The other model is the partial-thickness model that preserves the calcified cartilage; therefore, if the technique is performed correctly, it avoids the penetration of bone marrow cells inside the joint cavity 2.

Over time, many scoring systems have been created according to the needs of the study, including creation of *in vitro* and *in vivo* animal models 9-13. All existing scoring systems used to histologically evaluate the cartilage in animal models are based on full-thickness lesion repair and were adapted for use in the partial-thickness model, although they are not specific for this type of research. The present study aimed to describe a new semi-quantitative histological scoring system for use in experimental animal models of partial-thickness cartilage lesions.

## MATERIALS AND METHODS

### Experimental animal models

A partial-thickness lesion is a cartilage defect that does not reach the subchondral bone. Bleeding does not occur at the base of the lesion. The calcified layer is not removed. A total-thickness lesion is a deep cartilage defect that reaches the subchondral bone; in such cases, bleeding occurs at the base of the lesion.

A focal lesion (FL) is a partial- or total-thickness defect with normal cartilage surrounding the injury 14. It can predispose an individual to OA. OA is a cartilage lesion with a mirror lesion of the cartilage on the opposite articular surface, accompanied by a hypertrophic synovial membrane and degenerative changes of the meniscus and the subchondral bone 14,15.

### Sample preparation – Surgical procedure

In a previous study from our group 2, 30 knees were used from 15 female adult Dorper sheep (between 2 and 5 years of age). After general anesthesia, a lesion was made (10 mm in diameter) at the load area of the femoral medial condyle in each knee. All international, national, and institutional guidelines for the use of large animals were followed, and the local Animal Care and Use Ethical Committee approved the study (CEUA 1556-12) 2.

To avoid penetration of the subchondral bone and thermal lesions of the subjacent cartilage, drills were not used. Details of the technique were described previously 2.

The lesion was marked with a 10-mm punch usually employed for cutaneous biopsy. Subsequently, several longitudinal incisions were performed in the area with an 11-blade scalpel, and several perpendicular incisions were made to form a checkered pattern to weaken the cartilage. Using a bone curette, the cartilage was removed from the defect without removing the calcified layer. If no bleeding was observed, the procedure was considered to not have reached the subchondral bone.

After six months, the animals were euthanized. A trapezoidal fragment of the femoral medial condyle, containing the surgically created lesion, was removed with a manual saw. This block was immediately placed in conventional formalin solution and sent to the pathology laboratory.

### Fixation and Staining

In the laboratory, the blocks were fixed in a buffered formalin solution for 48 hours and then washed with distilled water and decalcified for 24 hours in a medium containing EDTA solution. Subsequently, the blocks were dehydrated in an ethanol series and finally fixed in paraffin.

A longitudinal axis was employed to cut several 5-μm sections, which were stained with hematoxylin & eosin (H&E), Safranin O and Masson's trichrome.

### New proposal

#### Unicamp Partial-thickness (UPT) score

Following and adapting the principles necessary to create an ideal cartilage histopathology score 16, the new system described here is simple, useful and exclusive for assessing experimental partial-thickness chondral lesions in animal models; it also has a detailed grading system.

The new scoring system is presented in Table 1.

#### Scoring items

The new score developed 2 has three main sections and a total of seven parameters. One section evaluates the reparative tissue formed inside the lesion, another assesses the native cartilage border (i.e., how it reacted to the lesion), and the final section evaluates the degenerative changes that may have occurred at the calcified cartilage and at the subchondral bone at the base of the lesion.

Each section has a subscore that is added to the total result. The score ranges from 0 to 21. A higher total indicates better formation of new cartilage. Each parameter is described below.

**Figure 1:**
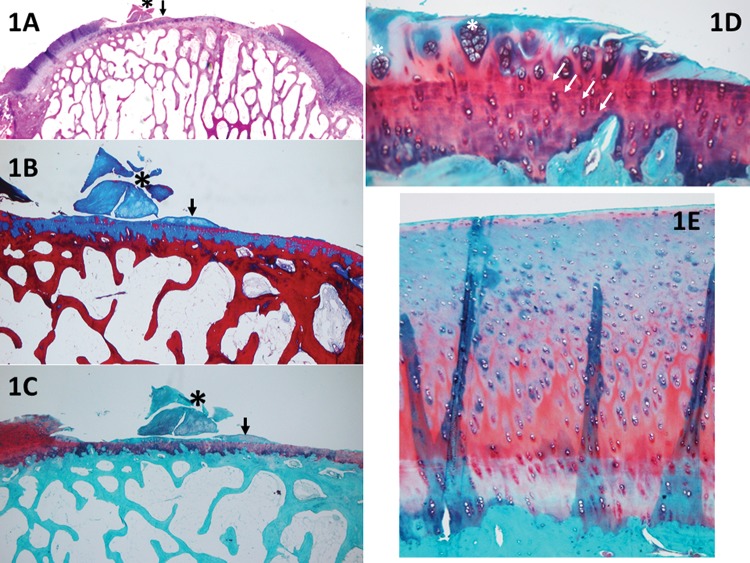
A panoramic view (1A, H&E) of a piece of cartilage that was removed from the sample (black asterisk), shown in detail with Safranin O stain (1C). With the Masson's trichrome stain (1B), it is possible to distinguish between old (red) and new (blue) collagen; therefore, it can be assumed that this piece of cartilage (stained in blue) resulted from cartilage growth. Nevertheless, the score cannot be applied because a discreet detachment occurred. Furthermore, in 1A-1C, it is possible to observe small cartilage growth (black arrows), which can be evaluated with the UPT score; the horizontal growth occupies less than 20% of the defect (1 point), and the vertical growth (at its highest point) corresponds to less than 20% of the border height (1 point). Panel 1D (Safranin O) shows an example of cartilage hypocellularity with >25% of clusters and few isolated chondrocytes. Most of the cellularity is formed by clusters (white asterisk). The white arrows indicate the line formed between the pre-existing cartilage and the new cartilage (e.g., a “cementing line”), called a “tidemark.” Panel 1E (Safranin O) shows an example of relatively normal cellularity, in which the new cartilage follows the pre-existing tissue pattern of chondrocyte distribution.

**Figure 2:**
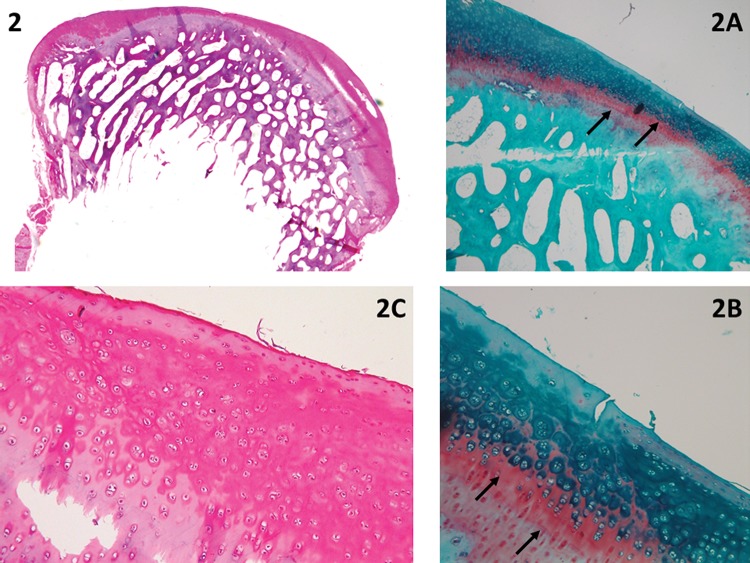
The complete regeneration of the cartilage is shown in a panoramic view. The difference between the new and old cartilage is clear due to the evident line that separates the section, similar to a “tidemark” separating the pre-existing and new tissue, as shown by Safranin O staining (arrows in 2A and 2B). The cellularity in the new cartilage has a distribution pattern similar to that of the old cartilage (2C). The following evaluation was made: horizontal filling completed 100% of the defect (4 points); vertical filling at its highest point was equal to the border height (100%, equal to 4 points); <25% of chondrocytes formed clusters (cellularity=1 point); Safranin staining was heterogeneous (1 point); the new tissue was integrated into both borders (2 points); and residual cartilage both at the base and the subchondral bone was intact and normal (3 and 4 points, respectively); total=19 points.

**Figure 3:**
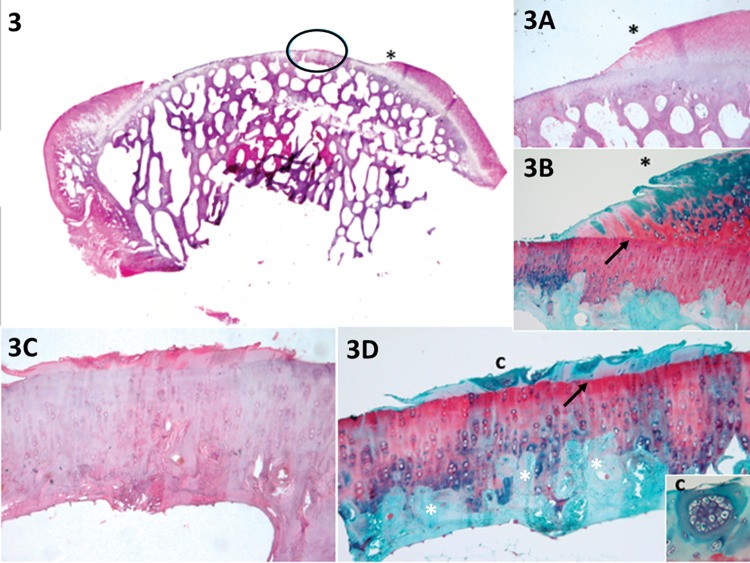
The panoramic view shows a small amount of growth of new cartilage in the middle of the defect (circled area). Panel 3A* shows new cartilage growth at the lateral internal border, as confirmed by Safranin O staining in 3B* (heterogeneous and with a tidemark – arrow). Panels 3C and 3D (circled area in 3) show little cartilage growth in the middle of the lesion. According to the evaluation, horizontal filling accounted for less than 20% of the defect (1 point); vertical filling at its highest point was less than 20% of the border height (1 point); the hypocellularity was mild with <25% of clusters (evident in 3B and 3D-c, 1 point); Safranin O staining was heterogeneous (1 point); the new tissue was integrated into only one border (1 point – 3*, 3A* and 3B*); and the residual cartilage and subchondral bone (white *) were normal (3 and 4 points, respectively), total=12 points.

**Figure 4:**
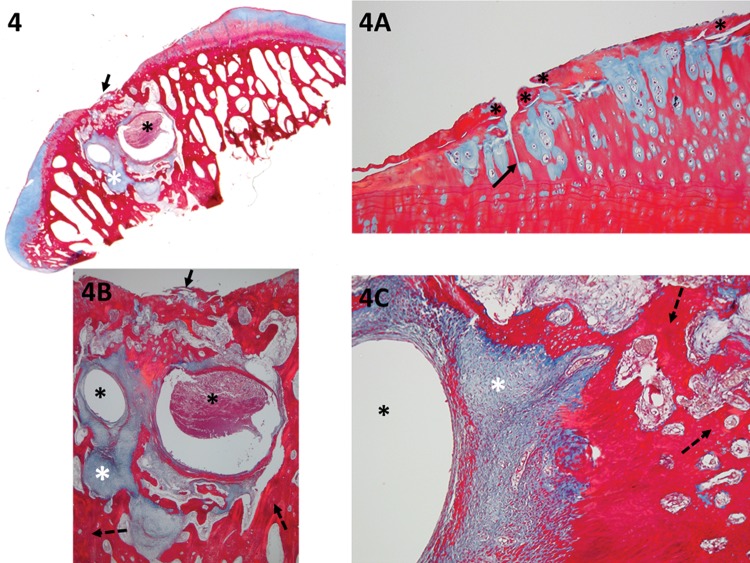
The panoramic view shows the exposition of the subchondral bone, likely due to an inadequate technique; therefore, the deep layers were not spared. The residual cartilage has fibrillation (black asterisk) and fissures (black arrow) (detail in 4A). Focal erosion is present (black arrow in 4 and 4B) (detail in 4B) (the lowest score should be considered in this case; thus, because of the presence of erosion, 1 point was assigned for the cartilage at the base of the lesion). The subchondral bone has a severe cystic lesion (black asterisk in 4, 4B and 4C) surrounded by loose, immature, fibrous connective tissue and new vessels (granulation tissue – white asterisk in 4, 4B and 4C). Because new sclerotic bone (detailed in 4B – dashed arrow) and remodeling bone formation (detailed 4C – dashed arrow) are observed, the total score for the subchondral bone is 1 point.

- New repair tissue inside the lesion
  1. Horizontal filling: employed to evaluate the degree of lesion closure, from the borders to the inside, i.e., how far the new tissue could grow from one of the lesion borders to the other. Greater growth in this direction indicates greater effectiveness in reducing the lesion size and protecting the underlying layers (Figure 1).
  2. Vertical filling: used to analyze the degree to which the lesion was filled, based on the border height. The highest point of the new tissue is measured (Figure 1).
  3. Cellularity: employed to evaluate the new cells (chondrocytes). The cellularity is “Normal” when the cell disposition is similar to the usual cartilage configuration. “Clusters” are groups of abnormal chondrocytes in the injured area. The chondrocytes (in a lacunar position) are nearer to the subchondral bone.
    At the superficial zone of the knee cartilage, the chondrocytes are normally isolated or in pairs 19. When the cartilage is injured, the remaining chondrocytes attempt to regenerate to compensate for the loss; however, this is not effective due to reduced irrigation and a decreased capacity for multiplication; therefore, the cells form clusters (i.e., the clustering process, groups of 2 or more chondrocytes with an abnormal nucleus and cytoplasm). During histological evaluation, chondrocyte clusters are used as indicators of cellular injury (Figure 1).
  4. Safranin staining (ECM): Safranin O stains glycosaminoglycans and is indicated for use in evaluating the quality of new and remaining cartilage matrix 17,18. “Homogeneous Safranin staining” indicates that the new cartilage is stained red, in the same manner as the surrounding tissue. “Heterogeneous Safranin staining” indicates that the stain is different from that of the surrounding cartilage, indicating areas with (stained red) and without glycosaminoglycans (stained green). “Negative” staining indicates no glycosaminoglycans on the matrix; therefore, nothing was stained in red (only in green) (Figure 2).
    Safranin O was used for red staining of the glycosaminoglycans in the ECM 2. The study that motivated the creation of the UPT score 2 also used Masson's trichrome to stain the collagen of the new tissue (ECM) in blue and the collagen of the remaining tissue in red. Masson's trichrome was also chosen to analyze the quality of the new matrix while using the main protein that composes the matrix (collagen) as a reference 17,18.
- Cartilage around the lesion
  This section evaluates the lateral internal borders of the lesion. This item is important for evaluation of the integration/junction between the new cartilage and the remaining cartilage so that the effectiveness of the intervention method can be evaluated. Good integration means that the new cartilage can be united with its surroundings and that the intervention was successful, with a decreased chance of evolving into a degenerative process.
  1. Border ingrowth or cartilage-to-cartilage integration: internal growth (ingrowth) on the lesion was analyzed (i.e., if the internal cartilage [new tissue] was connected to the lateral internal border of the lesion [surrounding cartilage]). In the partial-thickness model, centripetal ingrowth commonly occurs (growth from the borders to the center of the lesion) 17,18 (Figure 3).
- Degenerative changes
  If performed correctly, the partial-thickness model technique can spare the base of the lesion, leaving the residual cartilage intact, and the subchondral bone should not be impacted. To determine the adequacy of the intervention procedure, we examined the deeper layers. When they were integrated and normal, we assumed that the procedure was performed correctly. Furthermore, to determine the impact and evolution of the lesion, it is important to evaluate the residual cartilage and subchondral bone and to determine the degenerative changes listed below.
  1. Residual cartilage around the lesion: used to evaluate whether any degenerative change is present in the cartilage, such as “fibrillation or fissure”, that can evolve into focal erosion or severe disruption (Figure 4).
  2. Subchondral bone: used to analyze whether any degenerative changes occur to the subchondral bone. For example, “remodeling”, “sclerosis”, “callus” and “fracture necrosis” are local degenerative processes that can evolve into advanced degenerative joint disease (Figure 4).

## DISCUSSION

This article presents a new tool for subjective and semi-quantitative evaluation of repair tissue formed in partial-thickness articular cartilage lesions.

A suitable score for a partial-thickness model should evaluate the relevant features necessary to determine defect evolution, using the degenerative changes in the subchondral bone and surrounding cartilage as criteria to evaluate whether the technique was adequate and, regardless of whether repair was successful, analyze its cellularity and matrix 2.

The O'Driscoll score, described in 1988 9, is considered the gold standard for evaluation of cartilage regeneration in animal model studies because it uses the whole joint. Originally, it was divided into four subcategories: the nature of the tissue, structure, cellular degeneration at the lesion and cellular degeneration in the surrounding tissue. Since its publication, several modifications have been made, and other categories have been added. They are known as modified O'Driscoll scores.

The most important limitation of the O'Driscoll score and its modified versions is that they were created to evaluate lesions that perforate the subchondral bone; thus, they have an item that evaluates the reconstitution of the osteochondral junction, which is irrelevant to partial-thickness models. This same limitation holds for the Pineda score, Oswestry score and all subsequently published scores 8-12.

Other scores were developed to evaluate small biopsies in clinical studies, including the ICRS I and II 8,11,12. Due to the size of the evaluated fragment, some parameters cannot be evaluated, including “bonding with the adjacent cartilage and adjacent tissue.” Consequently, important information regarding the lesion and the surrounding tissue is lost.

The new score is relevant due to its utility in animal models that reproduce the most common human cartilage injury – partial-thickness tears 2; therefore, it may be useful for advancing research and impacting human treatment. This new tool was created to be used only in partial-thickness models. It evaluates cellularity, the matrix and degenerative changes in a simple manner.

Compared to existing tools, the difference is that this new tool does not assign points for positioning of the tidemark because the partial-thickness model should not include injury in the deep layers. Instead, this scale incorporates several parameters commonly used in pre-existing scales for OA assessment 8,11. The few existing partial-thickness animal model studies used inadequate scales designed for the full-thickness model or made adaptations to these scales, removing the evaluation of the subchondral bone 8-12. Mukoyama et al. 20 published an article about partial-thickness cartilage injury and used a histological score created by the modification of two previous scores, those of Pineda 10 and Wakitani 21; however, they evaluated only the repaired tissue inside the lesion but not its surroundings. Therefore, they could not compare the integrity between the new and the remnant tissue.

Our score is visual and has a detailed grading system that is useful for qualifying and quantifying an intervention, making it easier to compare results using the total score 16. The score is a subjective evaluation because it depends on the observer's experience for the evaluation of each section. Furthermore, it is semi-quantitative because it assigns points to each observed characteristic.

We propose to use this new scale for the evaluation of degenerative changes to subchondral bone as well as the calcified cartilage layer at the base of the lesion to evaluate the evolution of the defect and determine the quality of the technique used to create the lesion. A previous study by our group revealed that even a partial deep defect does not avoid injury to the subchondral bone 2. Furthermore, pressure overload upon an already fragile cartilage can cause increased injury.

There are some challenges to overcome. The UPT score was designed for an experimental animal model 2; therefore, it is necessary to remove the whole joint to study the evolution of the lesion and its surrounding tissue. Thus, the score cannot be used in clinical studies because a small biopsy would be insufficient for the proposed evaluation. It must still be validated, and its reproducibility must be proven.

Future studies are necessary to determine the correlation coefficient. These studies should evaluate the inter- and intra-observer variability of this histological score, comparing it to the gold-standard O'Driscoll score and determining its correlation with mechanical, biochemical, radiological, and clinical parameters. Our histological scale is comparable to other cartilage grading systems, and we expect little variability between various observers and similar variability during different periods; we also expect a more accurate analysis of partial-thickness samples than with other scores.

Our score provides a more accurate tool to evaluate the results of partial-thickness models, thereby increasing the quality of these studies and enhancing the scientific debate regarding intervention results.

This article emphasizes the importance of the partial-thickness animal model of cartilage repair and presents a new histological scoring system.

## AUTHOR CONTRIBUTIONS

Ponce MC wrote the manuscript and is the corresponding author. Zorzi AR and Amstalden MI developed the new score, oriented and organized the manuscript. Miranda JB revised the final version of the manuscript.

## Figures and Tables

**Table 1:** Unicamp Partial Thickness (UPT) Score.

A) New repair tissue inside the lesion	Feature	Score
Horizontal filling	75-100%	4
	50-74%	3
	20-49%	2
	1-19%	1
	0	0
Vertical filling	75-100%	4
	50-74%	3
	20-49%	2
	1-19%	1
	0	0
Cellularity	Normal	2
	Mild hipocellularity and/or <25% clusters	1
	Moderate to severe hipocellularity and/or >25%	0
	clusters	
Safranin staining	Homogenous	2
	Heterogenous	1
	Negative	0
B) Cartilage around the lesion	Feature	Score
Borders ingrowth or cartilage to cartilage integration	Bilateral	2
	Unilateral	1
	None	0
C) Degenerative changes	Feature	Score
Residual cartilage at the base of the lesion	Intact or integrated	3
	Fibrillation or fissure	2
	Focal erosion	1
	Severe disruption	0
Subchondral bone	Normal	4
	Mild cystic lesions or granulation tissue	3
	Moderate or severe cystic lesions or granulation tissue	2
	Remodelling, sclerosis, callus	1
	Fracture, necrosis	0
Total		0-21
